# Assessment of therapeutic outcome and role of reirradiation in patients with radiation-induced glioma

**DOI:** 10.1186/s13014-022-02054-x

**Published:** 2022-05-03

**Authors:** Makoto Ohno, Yasuji Miyakita, Masamichi Takahashi, Shunsuke Yanagisawa, Yukie Tamura, Daisuke Kawauchi, Miyu Kikuchi, Hiroshi Igaki, Akihiko Yoshida, Kaishi Satomi, Yuko Matsushita, Koichi Ichimura, Yoshitaka Narita

**Affiliations:** 1 Department of Neurosurgery and Neuro-Oncology, National Cancer Center Hospital, 5-1-1, Tsukiji, Chuo-ku, Tokyo, 104-0045 Japan; 2 Department of Radiation Oncology, National Cancer Center Hospital, 5-1-1, Tsukiji, Chuo-ku, Tokyo, 104-0045 Japan; 3 Department of Diagnostic Pathology, National Cancer Center Hospital, 5-1-1 Tsukiji, Chuo-ku, Tokyo, 104-0045 Japan; 4 Division of Brain Tumor Translational Research, National Cancer Center Research Institute, 5-1-1, Tsukiji, Chuo-ku, Tokyo, 104-0045 Japan; 5 Department of Brain Disease Translational Research, Faculty of Medicine, Juntendo University, 2-1-1 Hongo, Bunkyo-ku, Tokyo, 113-8421 Japan

**Keywords:** Radiation-induced glioma, *IDH1/2* mutations, Secondary neoplasms, Long-term survivors of malignancies, Reirradiation

## Abstract

**Background:**

We sought to clarify the optimal follow-up, therapeutic strategy, especially the role of reirradiation, and the diagnostic impact of *isocitrate dehydrogenase* (*IDH*) 1 and 2 mutation status in patients with radiation-induced glioma (RIG).

**Methods:**

We retrospectively reviewed the clinical characteristics and treatment outcomes of 11 patients with high-grade glioma who satisfied Cahan’s criteria for RIG in our database during 2001–2021. *IDH 1/2* mutations were analyzed by Sanger sequencing and/or pyrosequencing.

**Results:**

The RIGs included glioblastoma with *IDH 1/2* wild-type (n = 7), glioblastoma not otherwise specified (n = 2), anaplastic astrocytoma with *IDH1/2* wild-type (n = 1), and anaplastic astrocytoma not otherwise specified (n = 1). The median period from primary disease and RIG diagnosis was 17 years (range: 9–30 years). All patients underwent tumor removal or biopsy, 5 patients postoperatively received reirradiation combined with chemotherapy, and 6 patients were treated with chemotherapy alone. The median progression-free and survival times were 11.3 and 28.3 months. The median progression-free survival time of patients treated with reirradiation and chemotherapy (n = 5) tended to be longer than that of patients that received chemotherapy alone (n = 6) (17.0 vs 8.1 months). However, the median survival time was similar (29.6 vs 27.4 months). Local recurrence was observed in 5 patients treated with chemotherapy alone, whereas in 2 patients among 4 patients treated with reirradiation and chemotherapy. None of the patients developed radiation necrosis. In one case, the primary tumor was diffuse astrocytoma with *IDH2* mutant, and the secondary tumor was glioblastoma with *IDH 1/2* wild-type. Based on the difference of *IDH2* mutation status, the secondary tumor with *IDH 1/2* wild-type was diagnosed as a de novo tumor that was related to the previous radiation therapy.

**Conclusions:**

RIG can occur beyond 20 years after successfully treating the primary disease using radiotherapy; thus, cancer survivors should be informed of the long-term risk of developing RIG and the need for timely neuroimaging evaluation. Reirradiation combined with chemotherapy appears to be feasible and has favorable outcomes. Determining the *IDH1/2* mutational status is useful to establish RIG diagnosis when the primary tumor is glioma.

## Background

Radiotherapy is used for cancer treatments, including pediatric brain tumors and hematological malignancies, such as glioma, medulloblastoma, germ cell tumors, and leukemia. Despite an overall improvement in the survival rates of patients with these tumors, patients treated with radiotherapy are at risk of long-term neurological complications such as the development of progressive leukoencephalopathy, arteritis, hypopituitarism, and hypothalamic insufficiency [1]. One of the most serious late consequences of radiotherapy is secondary neoplasms, which occur in rare cases but represents a major cause of mortality in long-term survivors of childhood malignancies [2–6]. Among radiation-induced brain tumors, meningiomas and gliomas are the most frequently reported secondary neoplasms [1]. The cumulative risk of secondary brain tumors occurring after radiation therapy for pituitary adenomas is 2.0% at 10 years and 2.4% at 20 years, which is 10.5 times higher than that seen in the general population [3]. The cumulative risk of secondary brain tumors occurring among long-term acute lymphoblastic leukemia survivors is 0.8% at 10 years and 1.87% at 20 years [2].

Radiation-induced gliomas (RIGs) are typically high-grade tumors. The median latency period for developing RIGs is 8–11 years [4–6]. The overall standardized incidence ratio (SIR) for RIG in childhood cancer survivors is 10.8, and the SIR is different according to the follow-up period; 20.6 in 0–4 years follow-up, 7.5 in 5–9 years follow-up, 11.0 in 10–14 years follow-up, 12.5 in 15–19 years follow-up, 7.2 in 20–29 years follow-up, and 5.0 in 30 years follow-up [7]. The treatments of RIGs are usually challenging, and the clinical outcomes are generally poor [3–5, 8]. The median survival time (MST) of patients with RIGs is 11 months, with a 2-year survival rate of 20.2% [5]. Several studies and review articles have proposed a combination therapy of reirradiation (ReRT) and chemotherapy as a potential treatment option; however, there are few reports on the details of the combined therapy and their treatment outcomes. Thus, the optimal therapeutic approach for RIGs is not well defined [5]. Moreover, few studies investigated genetic alterations in RIGs [9–15], and their clinical impact remains unclear.

In this study, we retrospectively analyzed the clinical characteristics and treatment outcomes in 11 patients with RIG to clarify the optimal follow-up period from the treatment of the primary disease and therapeutic strategy, especially for the role of ReRT. We also investigated genetic alterations in 8 patients and evaluated the diagnostic impact of *isocitrate dehydrogenase* (*IDH*) 1 and 2 mutation status on establishing RIG diagnosis.

## Methods and materials

### Patient characteristics

This study was a retrospective observational study. We reviewed our departmental database between 2001 and 2021. We included patients who satisfied Cahan’s criteria, which were as follows: (1) the tumor must originate in a previously irradiated region (but not necessarily in the full-dose region), (2) there must be a sufficient latency time between irradiation and the onset of the postradiation tumor, (3) the tumor histology must be different from that of the primary tumor, and (4) the patient must not have pathologies that favor the development of tumors: Li-Fraumeni’s disease, von Recklinghausen’s disease, tuberous sclerosis, xeroderma pigmentation, or retinoblastoma [5, 16, 17].

The clinical, operative and radiological records of the patients were reviewed, and data on the following variables were collected: clinical and treatment history before RIG diagnosis, clinical and treatment history after RIG diagnosis, Karnofsky performance status (KPS) at the time of RIG diagnosis, presence or absence of comorbidities and leukoencephalopathy at the time of RIG diagnosis, date of operation for RIG, postoperative therapy for RIG, date of tumor recurrence of RIG, date of death or last hospital visit, the extent of resection of RIG, and treatment after tumor recurrence of RIG. The leukoencephalopathy was evaluated by magnetic resonance images (MRI) and graded based on the Common Terminology Criteria for Adverse Events version 5.0. The extent of resection of the RIGs was determined based on the surgeon’s operative notes and postoperative imaging studies and classified as follows: total if 100% of the contrast-enhanced lesion was resected; subtotal, if 95–99% of the lesion was resected; partial, if < 94% of the lesion was resected, or removed as a biopsy [18]. All patients were re-diagnosed by neuropathologists at our hospital according to the World Health Organization 2016 classification [19].

### Genetic analysis

Tumor DNA was extracted from frozen tumor tissues in 8 cases using a DNeasy Blood & Tissue Kit (Qiagen; Tokyo, Japan). The presence of hotspot mutations in the *IDH1* (R132) and *IDH2* (R172) genes was assessed by Sanger sequencing and/or pyrosequencing, as described previously [20, 21]. Pyrosequencing assays were designed to detect all known mutations in these genes [20]. The two mutation hotspots in the *telomerase reverse transcriptase* (*TERT*) gene promoter were analyzed in 8 tumors using Sanger sequencing and/or pyrosequencing, as reported previously [22]. The mutation hotspots at codons 27 and 34 of the histone H3.3 (*H3F3A*) gene, and those at codon 600 of the B-Raf (*BRAF*) gene, were analyzed in 6 tumors using Sanger sequencing and/or pyrosequencing [21]. The methylation status of the *O-6-methylguanine DNA methyltransferase* (*MGMT)* promoter was analyzed in 8 tumors using bisulfite modification of the tumor genomic DNA, followed by pyrosequencing, as previously described [22]. The *MGMT* promoter methylation status was defined as hypermethylation when its mean level at the 16 CpG sites was 16% and greater than 16%, and hypomethylation when less than 16% [18, 22].

### Statistical analysis

The latency period was defined as the interval between the date of diagnosis of the primary disease and that of RIG. Overall survival time (OS) was defined as the interval between the date of RIG surgery and death or the last follow-up, whichever occurred first. Progression-free survival time (PFS) was defined as the period between the date of RIG surgery and the detection of progression, death, or last follow-up. These times were calculated using the Kaplan–Meier method by JMP® ver. 15.1.0 software for Mac (SAS Institute Japan; Tokyo, Japan) and GraphPad Prism® ver. 9.2.0 for Mac (GraphPad Software; La Jolla, CA, USA).

## Results

### Patient characteristics of primary disease

We identified 11 patients who satisfied Cahan’s criteria and had RIG [5, 16, 17]. The patient characteristics of the primary disease are summarized in Table 1. The median age of the 11 patients was 12 years (range: 1–39 years), and there was no sex predominance (male: 6, female: 5). The primary diseases included germinomas (n = 2), acute lymphoblastic leukemias (n = 2), medulloblastomas (n = 3), diffuse astrocytoma with *IDH2* mutant (n = 1), pilocytic astrocytoma (n = 1), pituitary adenoma (n = 1), and a metastatic brain tumor from lung cancer (n = 1). All patients received cranial radiation. In Case 1, received continuous intraarterial bromodeoxyuridine combined with radiotherapy of 41 Gy in 23 fractions at 15-year-old and of 60 Gy in 34 fractions at 17-year-old [23]. The median latency time between the primary disease and RIG was 17 years (range: 9–30 years) (Table 1).

**Table 1:** Characteristics of patients with primary diseases

Case no.	Sex	Age at primary disease (years)	Primary disease	Location	Therapy		Chemotherapy	Latency (years)
					Radiation therapy	Radiation dose (Gy)		
1	M	15	Germinoma	Suprasellar	Local, Local^a^	41, 60^a^	Yes	30
2	M	25	Pituitary adenoma	Sellar	Local	60	No	20
3	M	1	Acute lymphoblastic lymphoma	Systemic	TB	18	Yes	9
4	M	20	Germinoma	Suprasellar	WB	50	Yes	13
5	F	12	Pilocytic astrocytoma	Hypothalamus	Local	54	No	22
6	F	9	Medulloblastoma	Cerebellum	CS	WB: 35.6, Local: 66, WS: 31.9	Yes	30
7	F	10	Medulloblastoma	Cerebellum	CS	WB: 40, Local: 60, WS: 30	Yes	13
8	M	2	Acute lymphoblastic lymphoma	Systemic	TB	12	Yes	15
9	F	15	Diffuse astrocytoma, *IDH2*-mutant	Left Frontal	Local	60	Yes	17
10	F	39	Metastatic brain tumor from Lung cancer	Multiple	Local, WB	CK: 22, WB: 30	Yes (TKI)	10
11	M	6	Medulloblastoma	Cerebellum	CS	WB: 23.4, Local: 55.8, WS: 23.4	Yes	22

*M* male, *F* female, *IDH* isocitrate dehydrogenase, *TB* total body, *CS* craniospinal, *WB* whole brain, *WS* whole spine, *CK* cyberknife, *TKI* tyrosine kinase inhibitor

^a^This patient received radiotherapy of 41 Gy in 23 fractions at 15-year-old and of 60 Gy in 34 fractions at 17-year-old

### Patient characteristics and treatment of RIGs

The characteristics of the 11 patients with RIG are summarized in Table 2. The median age of the patients was 34 years (range: 10–49 years). The RIGs included glioblastoma (GBM) with *IDH1/2* wild type (n = 7), GBM not otherwise specified (n = 2), anaplastic astrocytoma with *IDH1/2* wild type (n = 1), and anaplastic astrocytoma not otherwise specified (n = 1). All patients underwent tumor removal or biopsy and were diagnosed based on histopathological examination. Two patients had multiple intraparenchymal lesions (Case 3 and Case 6), and one had right cerebellar and pontine lesions with cerebrospinal dissemination (Case 4). Three patients showed leukoencephalopathy at the time of RIG diagnosis: Grade II in 2 patients and Grade I in 1 patient. Seven patients (63.6%) suffered from comorbidities, which were related to primary therapy: 3 had mild cognitive impairment, 2 had hypopituitarism, 2 had visual dysfunction, and 1 had short stature. No patient with leukoencephalopathy was associated with cognitive impairment. The median KPS at the time of RIG diagnosis was 80.

**Table 2:** Characteristics of patients with radiation-induced gliomas

Case no.	Sex	Age (years)	Secondary disease	Karnofsky performance status	Location	Leukoencephalopathy	Comorbidity	Initial treatment			Recurrent pattern	Treatment at first recurrence			PFS (months)	OS (months)	Status
								Operation	Chemotherapy	Radiation therapy		Operation	Chemotherapy	Radiation therapy			
1	M	45	GBM, NOS	80	Rt. insula	Grade I	Visual dysfunction	Partial removal	ACNU	No	Local	BSC			28.1	34.5	Dead
2	M	45	AA, NOS	70	Rt. temporal	No	No	Partial removal	TMZ	No	Local	BSC			3.0	8.4	Dead
3	M	10	GBM, *IDH1/2*-Wildtype	80	Lt. frontal Lt. parietal	No	Short stature	Biopsy	ACNU	Local RT 40 Gy/20fr	Local	No	Carboplatin, Etoposide	No	3.8	11.0	Dead
4	M	33	GBM, *IDH1/2*-Wildtype	60	Rt. Cerebellum Pons (dissemination)	Grade II	Hypopituitarism	Biopsy	Carboplatin, Etoposide	No	Dissemination	BSC			2.5	4.6	Dead
5	F	34	GBM, *IDH1/2*-Wildtype	80	Lt. temporal	No	Hypopituitarism Visual dysfunction	Total removal	TMZ	No	Local	Subtotal removal	TMZ	Local RT 45 Gy/25f	11.3	27.5	Dead
6	F	39	AA, *IDH1/2*-Wildtype	60	Rt. Parietal Rt. occipital	No	Mild cognitive impairment	Rt. parietal: partial removal Rt. occipital: partial removal	TMZ	No	Local	Rt. Parietal: Total removal Rt. Occipital: Total removal	TMZ	Local RT 60 Gy/30fr	8.8	27.3	Dead
7	F	23	GBM, *IDH1/2*-Wildtype	90	Rt. frontal	No	Mild cognitive impairment	Subtotal removal	TMZ	No	Local	No	TMZ	SRT 40 Gy/10fr	7.4	29.1	Dead
8	M	17	GBM, NOS	90	Rt. parietal	No	No	Total removal	TMZ	Local RT 66 Gy/33fr	Local	No	No	GKS	17.0	30.8	Dead
9	F	32	GBM, *IDH1/2*-Wildtype	80	Lt. frontal-parietal	Grade II	No	Biopsy	TMZ	Local RT 60 Gy/30fr	Distant	No	TMZ	SRT 42 Gy/7fr	23.0	35.1	Dead
10	F	49	GBM, *IDH1/2*-Wildtype	90	Rt. frontal	No	No	Total removal	TMZ, Bev	Local RT 50 Gy/25fr	Distant	Biopsy	Bev	SRT 42 Gy/7fr	15.9	28.3	Dead
11	M	28	GBM, *IDH1/2*-Wildtype	60	Lt. cerebellum	No	Mild cognitive impairment	Biopsy	TMZ, Bev	Local RT 40 Gy/15fr	No recurrence	No recurrence			9.8	9.8	Alive

*M* male, *F* female, *GBM* glioblastoma, *NOS* not otherwise specified, *AA* anaplastic astrocytoma, *IDH* isocitrate dehydrogenase, *Rt* right, *Lt* left, *ACNU* nimustine hydrochloride, *TMZ* temozolomide, *Bev* bevacizumab, *RT* radiation therapy, *BSC* best supportive care, *GKS* gamma knife radiosurgery, *SRT* stereotactic radiotherapy

For postoperative treatment, 5 patients received ReRT combined with chemotherapy, including ReRT/temozolomide (TMZ) (n = 2), ReRT/TMZ/bevacizumab (Bev) (n = 2), and ReRT/nimustine hydrochloride (ACNU) (n = 1); 6 patients were treated with chemotherapy alone, including TMZ (n = 4), ACNU (n = 1), and carboplatin and etoposide (n = 1). The ReRT regimens were as follows: 40 Gy in 15 fractions, 40 Gy in 20 fractions, 50 Gy in 25 fractions, 60 Gy in 30 fractions, and 66 Gy in 33 fractions.

Ten patients had tumor recurrences, and 7 patients received further treatments for recurrent tumors. All the patients were treated with chemotherapy. Four patients received ReRT at the initial location at the time of recurrence. Two patients with supratentorial tumors (Case 9 and Case 10) had tumor recurrence in the cerebellum at a distance from the initial location and received stereotactic radiotherapy consisting of 42 Gy in 7 fractions (Table 2).

### Treatment outcomes of RIGs

**Fig. 1:**
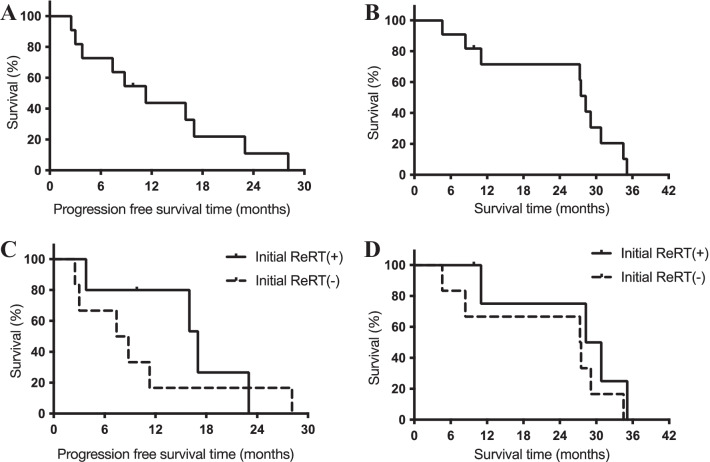
Kaplan–Meier curves of progression-free survival time (PFS) and overall survival time. **A** The median PFS was 11.3 months. **B** The median survival time was 28.3 months. **C** The median PFS in patients treated initially with reirradiation (ReRT) combined with chemotherapy (n = 5) was 17.0 months, and that in patients receiving chemotherapy alone was 8.1 months (n = 6). **D** The median survival times of patients treated initially with ReRT combined with chemotherapy (n = 5) was 29.6 months, and that in patients receiving chemotherapy alone (n = 6) was 27.4 months

The median PFS and median survival time (MST) in 11 patients with RIG were 11.3 months and 28.3 months, respectively (Fig. 1A, B). The median PFS in patients initially treated with ReRT combined with chemotherapy (n = 5) was 17.0 months; this was longer than that of patients treated with chemotherapy alone (8.1 months, n = 6) (Fig. 1C). The MST in patients initially treated with ReRT combined with chemotherapy (n = 5) and those receiving chemotherapy alone (n = 6) were 29.6 and 27.4 months (Fig. 1D).

The tumor recurrence pattern after initial treatment for RIGs was evaluated by radiological examinations in 9 patients, excluding 1 patient who presented with cerebrospinal dissemination and 1 patient who did not have a recurrence. Among 4 patients treated initially with ReRT combined with chemotherapy, 2 had local recurrence, and 2 had distant recurrence; all 5 patients treated with chemotherapy alone had local recurrence.

None of the patients was observed to develop symptomatic radiation necrosis, which could be caused by a high cumulative radiation dose during the follow-up period.

### Genetic alterations of RIGs

The genetic alterations of 8 patients whose tumor samples were available for analysis are summarized in Table 3. There were no alterations in the *IDH1/2* or *TERT* promoters in the 8 cases, and no *BRAF* or *H3F3A* mutations were found in the 6 cases for which data was available. Two tumors had hypermethylated *MGMT* promoters, whereas the other six had hypomethylated promoters.

**Table 3:** Summary of genetic alterations in radiation-induced gliomas

Case no.	Secondary disease	*IDH1/2*	*BRAF*	*H3F3A*	*TERT*	*MGMT*
3	GBM, *IDH1/2* Wild-type	WT	ND	ND	WT	Hypomethylation
4	GBM, *IDH1/2* Wild-type	WT	WT	WT	WT	Hypomethylation
5	GBM, *IDH1/2* Wild-type	WT	WT	WT	WT	Hypomethylation
6	AA, *IDH1/2* Wild-type	WT	WT	WT	WT	Hypermethylation
7	GBM, *IDH1/2* Wild-type	WT	WT	WT	WT	Hypomethylation
9	GBM, *IDH1/2* Wild-type	WT	ND	ND	WT	Hypomethylation
10	GBM, *IDH1/2* Wild-type	WT	WT	WT	WT	Hypomethylation
11	GBM, *IDH1/2* Wild-type	WT	WT	WT	WT	Hypermethylation

*GBM* glioblastoma, *AA* anaplastic astrocytoma, *WT* wild-type, *ND* not determined, *IDH* isocitrate dehydrogenase, *BRAF* B-Raf, *H3F3A* histone H3.3, *TERT* telomerase reverse transcriptase, *MGMT* O-6-methylguanine DNA methyltransferase

### Illustrative cases

We presented 2 illustrative cases; one case showed the favorable therapeutic effect of ReRT/TMZ/Bev (Case presentation 1: Case 11), and the other case showed the usefulness of *IDH1/2* mutational status evaluation in establishing the RIG diagnosis (Case presentation 2: Case 9).

### Case presentation 1

**Fig. 2:**
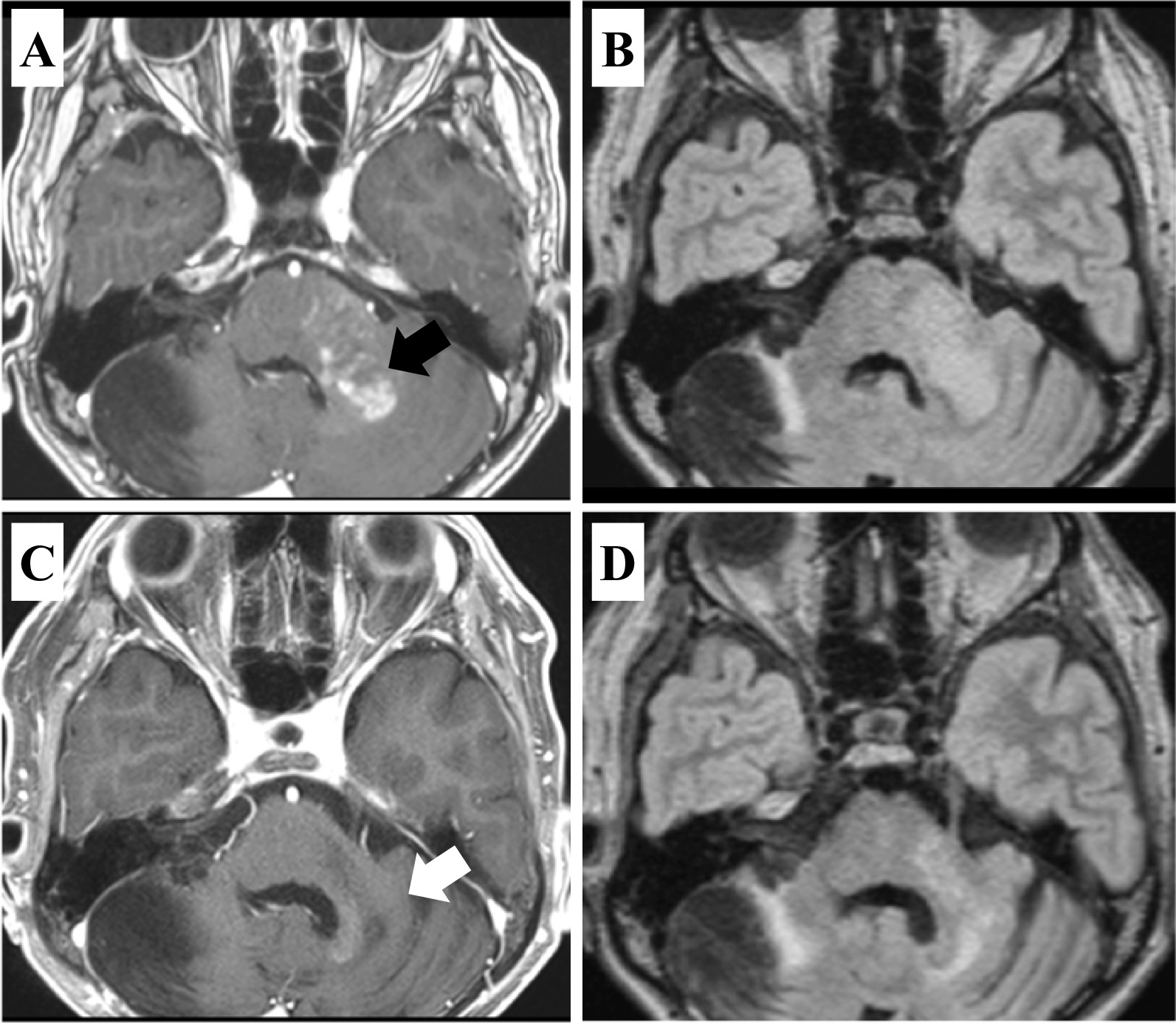
Representative patient treated with reirradiation, temozolomide, and bevacizumab (ReRT/TMZ/Bev) showing a favorable response (Case 11). **A** Preoperative T1-weighted magnetic resonance image with gadolinium enhancement and **B** fluid-attenuated inversion recovery (FLAIR) image showing an enhanced tumor in the left cerebellum (black arrow). **C** T1-weighted magnetic resonance image with gadolinium enhancement and **D** FLAIR image obtained 9.8 months after ReRT/TMZ/Bev treatment showing a favorable response (white arrow)

A 6-year-old boy (Case 11) initially presented with headache, vomiting, and conscious disturbance and underwent total removal of a right cerebellar tumor. The tumor was diagnosed as a medulloblastoma, and combined chemotherapy was performed with craniospinal radiation of 23.4 Gy in 13 fractions and local radiation up to 55.8 Gy in 31 fractions. Twenty-two years after the treatment for medulloblastoma, the patient presented with dizziness; an MRI revealed a left cerebellar contrast-enhanced lesion (Fig. 2A, B). He underwent a biopsy and was diagnosed as having GBM with *IDH1/2* wild-type. He received ReRT at a dose of 40 Gy in 15 fractions combined with TMZ/Bev and maintenance TMZ/Bev therapy. The tumor showed a complete response, and the patient did not develop tumor recurrence 9.8 months after the treatment for GBM with *IDH1/2* wild-type (Fig. 2C, D).

### Case presentation 2

**Fig. 3:**
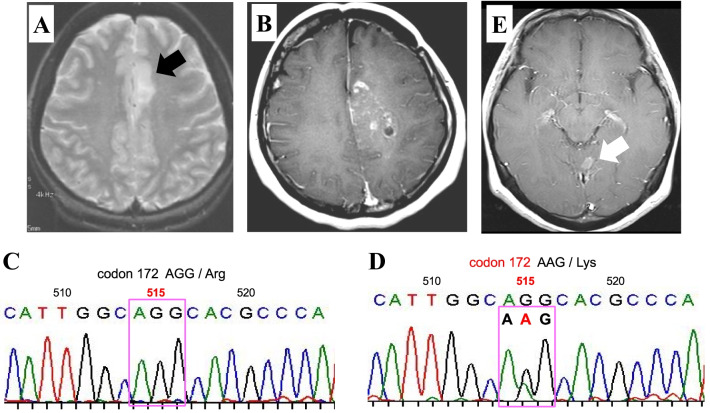
Representative patient treated with reirradiation and temozolomide (ReRT/TMZ) showing the usefulness of *IDH1/2* mutational status evaluation in establishing the RIG diagnosis (Case 9). **A** T2-weighted magnetic resonance image at the initial presentation showing a hyperintense lesion in the left medial frontal lobe (black arrow). **B** T1-weighted magnetic resonance image with gadolinium enhancement obtained 17 years after the primary tumor showing a contrast-enhanced lesion just posterior to the primary tumor, which was included within the prior radiation field. **C** Sanger sequencing analysis of the secondary tumor (glioblastoma) showing the homozygous G nucleotide at codon 515 of the *IDH2* gene, indicating the *IDH2* gene was wild-type. **D** Sanger sequencing analysis of the primary tumor (diffuse astrocytoma) showing the heterozygous G and A nucleotides at codon 515 of the *IDH2* alleles, indicating the *IDH2* gene was mutant. **E** T1-weighted magnetic resonance images with gadolinium enhancement were obtained 23.0 months after the secondary tumor diagnosis showing a distant recurrence in the cerebellum (white arrow)

A 12-year-old female patient (Case 9) initially presented with a cataplectic attack; 3 years later, an MRI exam revealed a left frontal non-contrast-enhanced tumor (Fig. 3A). She underwent subtotal resection and was diagnosed with diffuse astrocytoma. After the operation, she received radiation therapy at a dose of 60 Gy in 30 fractions and chemotherapy with ACNU. Seventeen years after treatment for the diffuse astrocytoma, she developed a contrast-enhanced lesion just posterior to the primary tumor, which was included within the prior radiation field (Fig. 3B). She underwent a biopsy, and the secondary tumor was diagnosed as GBM with *IDH1/2* wild-type (Fig. 3C). We performed Sanger sequencing analysis of the *IDH1/2* gene in the primary tumor and found that the tumor had an *IDH2* mutation (Fig. 3D). Because *IDH1/2* mutations maintain through tumor recurrence [24], the secondary tumor with *IDH1/2* wild-type was no recurrence from the primary tumor with *IDH2* mutation but was a de novo tumor that was most likely to be related to the previous exposure. Therefore, we diagnosed the secondary tumor as RIG. The patient received ReRT at a dose of 60 Gy in 30 fractions combined with TMZ; however, the patient had a distant recurrence in the cerebellum 23.0 months after the treatment for GBM with *IDH1/2* wild-type (Fig. 3E) and died 12.1 months thereafter.

## Discussion

In this study, we observed a median latency time of 17 years, with a range of 9 to 30 years. Among the 11 patients with RIG, ReRT combined with chemotherapy was performed in 5 patients at the initial treatment of RIG and for 6 patients at the time of recurrence; the median PFS and MST were 11.3 months and 28.3 months, respectively. Local recurrence was observed in all 5 patients initially treated with chemotherapy alone, whereas in 2 patients among 4 patients treated initially with ReRT combined with chemotherapy. We identified no genetic alterations in the *IDH1/2* and *TERT* promoters or in the *H3F3A* and *BRAF* genes. Moreover, we found that the *IDH1/2* mutational status evaluation helped establish RIG diagnosis in cases whose *IDH1/2* mutational states differed between the primary and secondary glioma.

The optimal screening frequency or follow-up time of childhood cancer survivors remains unclear [25]. Previous studies reported that the median latency period was 8–11 years, and the incidence of RIG largely disappeared after 15–20 years [4–6]. However, in our cohort, the median latency time from the primary cancer treatment to the development of RIG was 17 years, with a range of 9 to 30 years, and 5 out of 11 patients (45.5%) had a latency period of 20 years or more. Nakao et al. also reported that the latency period was more than 20 years in 4 patients [13]. The French Childhood Cancer Survivor Study showed that a latency period of more than 25 years was observed in 25 (53.2%) patients among 47 patients with RIG [26]. These results indicate that pediatric patients with primary diseases treated successfully with radiation therapy have a risk of developing RIG more than 20 years after the initial treatment. Regular imaging surveillance is not recommended due to financial and emotional stress, rarity of incidence and lack of evidence that early identification of RIG could improve outcome [27]. However, based on our results, we conclude it is important to inform cancer survivors about the long-term risk of developing RIG beyond 20 years and the need for timely neuroimaging evaluation when they present neurological symptoms.

ReRT is a primary treatment option in RIG management [4, 5]. Paulino et al. reported that patients who underwent ReRT for RIG showed better survival rates than those who did not (13 vs. 8 months; *p* = 0.0009), suggesting that ReRT was efficacious in treating these tumors [4]. Yamanaka et al. reported that the MST of patients who received surgery, chemotherapy, and ReRT was 18 months, whereas the remainder of patients who did not receive combined modality therapy had an MST of 9 months (*p* = 0.0006), suggesting that the combination of ReRT and chemotherapy is a potentially effective treatment option for RIG [5]. In our study, the median PFS and MST were 11.3 and 28.3 months, better than those reported in previous studies [4, 5, 28]. We also showed that patients initially receiving ReRT combined with chemotherapy tended to have a longer PFS and more favorable local control than those initially receiving chemotherapy alone. These are key findings, as they suggest the potential effect of initial ReRT combined with chemotherapy on local tumor control and emphasize the importance of ReRT in RIG treatment.

A serious concern of ReRT in the treatment of RIG is the risk of radiation necrosis. Fetcko et al. reported that 5.9% of patients developed radiation necrosis, and 3.3% had major neurological deficits after stereotactic radiosurgery (SRS) treatment for recurrent high-grade gliomas [29]. Shanker et al*.* also reported that the radiation necrosis rates after ReRT for recurrent high-grade gliomas were 7.1% for fractionated stereotactic radiotherapy, 6.1% for SRS, and 1.1% for conventional radiotherapy [30]. Paulino et al. reported from literature reviews that the risk of developing necrosis is less than 10% in the patients who underwent ReRT treatment for RIG [4]. In this study, we did not observe radiation necrosis in any patient. One possible reason for the low rate of radiation necrosis is that the period between the first and second radiation sessions is usually more than 10 years, and most patients with RIG die within 3 years; therefore, late complications related to ReRT might not be clinically relevant. To minimize the risk of radiation necrosis, the addition of Bev to ReRT may be a promising option. Cuneo et al. observed radiation necrosis in 19% of patients who received SRS without bevacizumab, and in 5% of those who received SRS with Bev, indicating Bev may reduce the risk of developing radiation necrosis [31]. The two patients (Cases 10 and 11) received Bev combined with postoperative ReRT and TMZ, and they did not develop symptomatic radiation necrosis during the follow-up period with 28.3 and 9.8 months. In addition, as shown in Case 11, ReRT combined with TMZ/Bev could have significant therapeutic effect (Fig. 2). It will be of interest to investigate the efficacy of combined ReRT and TMZ/Bev therapy in larger patient cohort with RIG.

Treatment-related comorbidities might influence the management and make the treatment challenging in patients with RIG. We found 7 patients (63.6%) who had comorbidities, which were related to primary therapy. Family support was needed to safely perform chemotherapy in patients with mild cognitive impairment or visual dysfunction and to maintain hormone replacement in those with hypopituitarism. In treating patients with these comorbidities, careful monitoring is mandatory to avoid treatment-related complications.

We investigated genetic alterations in 8 patients whose tumor samples were available. We found no alterations in the *IDH1/2* or *TERT* promoters or in the *H3F3A* or *BRAF* genes. Two patients had a hypermethylated *MGMT* promoter; the other 6 patients had a hypomethylated *MGMT* promoter. These results are consistent with those of previous reports and confirm the genetic characteristics of RIG [10–15, 32]. Recent comprehensive molecular analyses revealed that RIGs had recurrent *PDGFR* amplification, loss of *CDKN2A/B* and absence of histone 3 and *IDH1/2* mutations and also showed that their DNA methylation patterns closely resembled those of sporadic pediatric GBM RTK1 tumors [14, 15, 32]. These observations suggest that RIGs are molecularly distinct from adult diffuse gliomas and aberrant activation of the MAPK/ERK pathway together with loss of cell cycle control facilitates the tumorigenesis of RIG [14, 15, 32].

To establish the diagnosis of RIG, the tumor histology of the secondary tumor must be different from that of the primary tumor [5, 16, 17]. However, when the primary tumor is diffuse glioma, differentiating the secondary tumor from the recurrence of the primary tumor or de novo tumor that was related to the previous radiation exposure is difficult. In that situation, if the *IDH1/2* mutational status is different between the primary and the secondary tumors, the tumor origin is thought to be different between these two tumors. Thus, the *IDH1/2* mutational status difference between the primary and secondary tumors could help differentiate the secondary tumor from the recurrence of the primary or de novo tumors. In Case 9, the primary tumor had *IDH2* mutation, and the secondary tumor did not. Based on the difference in the *IDH1/2* mutational states between the primary and secondary tumors, we could conclude that secondary tumor with *IDH1/2* wild-type did not develop from the primary tumor with *IDH2* mutation, but the secondary tumor was a de novo tumor that was related to the previous radiation therapy. Furthermore, the observation that secondary tumor had *IDH1/2* wild-type was consistent with prior studies that RIGs do not harbor *IDH1/2* mutations [12–14]. We recommend evaluating the *IDH1/2* mutational status between the primary and secondary tumors when the primary tumor was glioma.

Our study had certain limitations. First, this was a retrospective study, and the indications or dose/fraction regimens of ReRT were heterogeneous. The indications or dose/fraction regimens of ReRT might have depended on the previous radiation field or regimen or period from the previous radiation; thus, heterogeneity was inevitable. Second, we did not investigate the genetic status in three patients because tissue samples were unavailable. Thus, further studies are needed to elucidate the genetic characteristics of RIG. Third, our cohort was too small to draw definitive conclusions. We acknowledge that the power of the survival analysis regarding the usefulness of ReRT, and the ReRT-related toxicity, was limited by the sample size; therefore, our results need to be confirmed in larger cohort studies.

## Conclusions

RIG can occur beyond 20 years after successful treatment of the primary disease using radiotherapy; thus, cancer survivors should be informed of the long-term risk of developing RIG and the need for timely neuroimaging evaluation when they present neurological symptoms. ReRT combined with chemotherapy appears to be feasible and has favorable outcomes. ReRT combined with TMZ/Bev could be a promising therapeutic approach. Determining the *IDH1/2* mutational status is useful to establish RIG diagnosis when the primary tumor is glioma.

## Data Availability

The datasets used and/or analyzed during the current study are available from the corresponding author upon reasonable request.

## Abbreviations

RIG: Radiation-induced glioma
ReRT: Reirradiation
GBM: Glioblastoma
TMZ: Temozolomide
Bev: Bevacizumab
ACNU: Nimustine hydrochloride
IDH: Isocitrate dehydrogenase
OS: Overall survival
MST: Median survival time
PFS: Progression-free survival time
MGMT: O-6-methylguanine DNA methyltransferase
TERT: Telomerase reverse transcriptase
BRAF: B-Raf
H3F3A: Histone H3.3
MAPK/ERK: Mitogen-activated protein kinase/extracellular signal-regulated kinase
